# Challenges of Developing Robust AI for Intrapartum Fetal Heart Rate Monitoring

**DOI:** 10.3389/frai.2021.765210

**Published:** 2021-10-26

**Authors:** M. E. O’Sullivan, E. C. Considine, M. O'Riordan, W. P. Marnane, J. M. Rennie, G. B. Boylan

**Affiliations:** ^1^ INFANT Research Centre, University College Cork, Cork, Ireland; ^2^ Department Obstetrics and Gynaecology, University College Cork, Cork, Ireland; ^3^ School of Engineering, University College Cork, Cork, Ireland; ^4^ Institute for Women’s Health, University College London, London, United Kingdom; ^5^ Department of Paediatrics and Child Health, University College Cork, Cork, Ireland

**Keywords:** cardiotocography (CTG), fetal heart rate (FHR), hypoxic ischaemic encephalopathy (HIE), labour, pregnancy, fetal hypoxia, artificial intelligence, machine learning

## Abstract

**Background:** CTG remains the only non-invasive tool available to the maternity team for continuous monitoring of fetal well-being during labour. Despite widespread use and investment in staff training, difficulty with CTG interpretation continues to be identified as a problem in cases of fetal hypoxia, which often results in permanent brain injury. Given the recent advances in AI, it is hoped that its application to CTG will offer a better, less subjective and more reliable method of CTG interpretation.

**Objectives:** This mini-review examines the literature and discusses the impediments to the success of AI application to CTG thus far. Prior randomised control trials (RCTs) of CTG decision support systems are reviewed from technical and clinical perspectives. A selection of novel engineering approaches, not yet validated in RCTs, are also reviewed. The review presents the key challenges that need to be addressed in order to develop a robust AI tool to identify fetal distress in a timely manner so that appropriate intervention can be made.

**Results:** The decision support systems used in three RCTs were reviewed, summarising the algorithms, the outcomes of the trials and the limitations. Preliminary work suggests that the inclusion of clinical data can improve the performance of AI-assisted CTG. Combined with newer approaches to the classification of traces, this offers promise for rewarding future development.

## 1 Introduction

Ensuring the safe passage of a baby through the birth canal remains a major challenge globally. Despite improvements in stillbirth and neonatal mortality rates, intrapartum-related hypoxia (“birth asphyxia”) is estimated to contribute to almost a quarter of the world’s annual 3 million neonatal deaths and almost a half of the 2.6 million third trimester stillbirths (Lee et al., 2013). The WHO estimated in 2005 that as many as 1 million survivors of birth asphyxia may develop cerebral palsy, learning difficulties or other disabilities each year. In England, the 2019/20 annual report of NHS Resolution (NHSR), the body that oversees clinical negligence claims, stated that £2.3 billion was spent on clinical negligence payments, of which 50% went on settling obstetric claims (which represented just 9% of the total claims made). NHSR estimated that for every baby born in England £1100 was paid in indemnity costs (NHS Resolution, 2019).

Currently, the only non-invasive way of assessing the fetus in labour is by monitoring fetal heartrate. Cardiotocography (CTG) is a technique that measures changes in fetal heart rate (FHR) and relates it to uterine contractions (UC) in order to identify babies who are becoming short of oxygen (hypoxic). CTG monitoring was introduced in the 1960s despite the absence of RCTs. Since then, a Cochrane review of 13 trials involving 37,000 women has shown that continuous CTG monitoring compared to intermittent auscultation was associated with a 50% reduction in neonatal seizures (Alfirevic et al., 2017). The review was dominated by the large Dublin trial which enrolled 12,964 women in 1981–1983 (MacDonald et al., 1985). This trial showed no difference in neonatal mortality or cerebral palsy rates. Many guidelines and textbooks on CTG interpretation have been published over the years, the most recent being the NICE intrapartum care guideline of 2014, updated in 2017 (National Institute for Health and Care Excellence (NICE), 2017). CTG interpretation is heavily dependent on pattern recognition, in particular the FHR response to UCs. Abnormal patterns, such as “late” decelerations, can indicate fetal hypoxia, but the CTG is an overly sensitive test; 60% of babies born after their CTG showed such changes were not acidotic (Beard and Finnegan, 1974). CTG interpretation has low inter- and intra-observer agreement rates, and even experts can differ in their interpretation of the same CTG.

The potential of CTG monitoring has not been realised in spite of major efforts aimed at training staff. NHSR has conducted several reviews (10 years of maternity claims (NHS Resolution, 2018) and 5 years of cerebral palsy claims (NHS Resolution, 2017). Errors with the interpretation of FHR monitoring was the most common theme and were often related to systemic and human factors. Uninterpretable CTGs were also common, with a wait and see approach being taken when there was possible loss of contact. The Royal College of Obstetricians & Gynaecologists (RCOG) “Each Baby Counts” report reached the same conclusion (Royal College of Obstetricians and Gynaecologists, 2020). The latest NHSR review recommended that CTG interpretation should not occur in isolation, but as part of a holistic assessment.

With artificial intelligence (AI), we can now take a fresh, unbiased look at the CTG. Previous attempts at using AI analysis of CTG have not proved successful. Most aimed to mimic human methods of analysis (e.g. recognition of FHR baseline, FHR variability and decelerations). However, modern computer systems using more advanced machine learning methods can include wide ranging analysis. AI systems are available 24/7, and are not affected by human factors such as fatigue, distraction, bias, poor communication, cognitive overload, or fear of doing harm. All of these were identified as limiting factors by the RCOG “Each Baby Counts” reports. Better ways of using and interpreting the CTG have the potential to reduce death and disability, and to prevent significant litigation costs.

## 2 Review of Prior Art in AI for CTG

### 2.1 Algorithms Used in Randomised Control Trials

Recent systematic reviews of AI for CTG concluded that prior studies did not manage to improve rates of neonatal acidosis, seizures, death, unnecessary interventions or ICU admissions (Campanile et al., 2018; Balayla and Shrem, 2019; Garcia-Canadilla et al., 2020). One study found that inter-rater reliability between humans and AI was moderate but that AI models that mimic human interpretation is akin to adding a “second evaluator with similar instructions” (Balayla and Shrem, 2019). This suggests that for decision support to be effective, it should add value through features that are not obvious to the human. The three RCTs included in the review paper, which are the only trials that compare human and AI CTG interpretation, are revisited below. The three systems used hand-crafted features that generally aimed to replicate the International Federation of Gynecology and Obstetrics (FIGO) guidelines (Ayres-de-Campos et al., 2015).

The INFANT (Intelligent Fetal AssessmeNT) system was developed over 20 years ago to extract and quantifies the following FHR features: signal quality, baseline, variability, accelerations, decelerations and their timing in relation to contractions. These are the features that are typically interpreted by the human in current clinical practice. The INFANT system extracts these features using numerical algorithms and artificial neural networks (Keith and Greene, 1994). Relevant clinical information, including cervical dilation, analgesia, fetal blood sampling and risk factors (intra-uterine growth restriction, placenta abruption and meconium) are also considered in the AI model. The system uses over 400 rules to interpret the data and provide decision support. It does not provide any recommendations for actions that should be taken in response to detected FHR abnormalities (Keith and Greene, 1994).

A multicentre RCT of this system on 47,000 patients was completed in 2017, which found that the decision-support software did not improve clinical outcomes, despite its effectiveness in correctly detecting FHR abnormalities (Brocklehurst et al., 2017). The hypotheses that substandard care was due to failure to identify non-reassuring CTG and that a decision-support system would reduce unnecessary interventions were not supported. The study suggests that substandard care was due to management decisions after identifying CTG abnormalities. The decision-support system used in the trial did not include clinical information pertaining to the labour (i.e. labour duration and progress). Including this information in the decision support system may have improved decisions to escalate.

Omniview-SisPorto 3.5 provides alerts based on computer analysis of CTG. It classifies CTG into four classes (reassuring, non-reassuring, very non-reassuring and pre-terminal) based on FIGO guidelines (Ayres-de-Campos et al., 2015), including definitions of late/prolonged/repetitive decelerations, reduced variability and baseline variation (Ayres-de-Campos et al., 2008). Their preliminary results showed that the agreement percentage between human and computer classification of contractions, accelerations and decelerations was 87, 71 and 68%, respectively (Costa et al., 2010). An RCT of the system on 7,320 patients was recently conducted (Nunes et al., 2017). The study concluded that while very low rates of acidosis were observed, the reduction in the rates of acidosis and obstetric interventions between the two arms of the study were not statistically significant.

A smaller RCT was conducted on a quantitative cardiotocography (qCTG) decision-support system, which enrolled 720 patients (Ignatov and Lutomski, 2016). The qCTG system computes features based on three domains: FHR, FHR micro-fluctuations, and decelerations. The features derived from FHR micro-fluctuations are the extrema per minute, the mean beat-to-beat variability per minute, and the oscillation amplitudes. A score of 0–6 is calculated for each domain and summed, giving an overall score of 0–18. The primary outcomes of the trial were hypoxia, acidemia, caesarean section and forceps extraction. Secondary outcomes were 5-minute Apgar, neonatal seizures and NICU admission. Reduced risks were observed for all outcomes in the interventional arm compared to the control arm. However, due to the small sample size of this study, a larger RCT is required to validate these findings.

### 2.2 Alternative Approaches

The aforementioned RCTs used computer-based algorithms that were largely based on features defined by the thresholds for baseline, variability and decelerations in the FIGO guidelines. Alternative approaches have been investigated which provide AI-based CTG interpretation in a manner that applies feature engineering theory from other domains that may complement existing human interpretation. While such systems have not yet been validated in RCTs, preliminary results are promising.

A control theory approach has been proposed to model the dynamic relationship between FHR and UC as an impulse response function (IRF) (Warrick et al., 2009). Pairing FHR and UC as an input-output system is clinically relevant, as decelerations are classified in response to the contractions. Early decelerations coincide with contractions, and do not indicate fetal hypoxia or acidosis. Late decelerations occur more than 20 s after a contraction and are indicative of hypoxia. Prolonged decelerations span multiple contractions and are indicative of hypoxia (Ayres-de-Campos et al., 2015). This method showed that IRFs in pathological cases resulted in longer delays between contractions and corresponding decelerations. IRF, FHR baseline and FHR variability were used as input features to a support vector machine (SVM) to classify normal and pathological CTGs. The training dataset consisted of 189 normal outcome cases and 31 pathological outcome cases. Their definition of a pathological case was death, or evidence of hypoxic ischemic encephalopathy (HIE), or a base deficit of more than 12 mmol per litre (mmol/L) meaning an acidic pH. The SVM correctly detected 50% of pathological cases with a false positive rate of 7.5% (Warrick et al., 2009).

A method using phase-rectified signal averaging to compute the mean decelerative capacity (DC) of FHR has been proposed (Georgieva et al., 2014). DC was compared to short-term variability (STV), which is considered a strong indicator of pH and has been used in previous studies. The results showed that DC predicted acidemia with 0.665 Area under the Curve (AUC). By comparison, STV achieved 0.606 AUC. Correlation between DC and STV was low, indicating that both may be used in multivariate analysis for improved prediction.

The FHR frequency content can be segmented in to low-frequency (0.04–0.15 Hz), mid-frequency (0.15–0.5 Hz) and high-frequency (0.5–1.0 Hz) bands. These bands correspond to sympathetic activity, fetal movement, and fetal breathing, respectively. The spectral densities and ratios between bands have been previously used to classify normal and pathological CTGs (Signorini et al., 2003; Spilka et al., 2013; Zhao et al., 2018). Fractal analysis and the Hurst parameter have been shown to be a robust alternative to using arbitrarily defined frequency bands, and predicted fetal acidosis with an AUC of 0.87 (Doret et al., 2015).

CTG is a very dynamic signal and the evolution of the CTG toward delivery is significant. An approach described in (Dash et al., 2014) segments the full CTG record into much shorter segments, extracts features and thus represents each full CTG record as a sequence of feature values, which are used as input to a Bayesian classifier. This method achieved a true negative rate (TNR) and true positive rate (TPR) of 0.817 and 0.609, respectively, outperforming SVM models trained on the same dataset.

**FIGURE 1:**
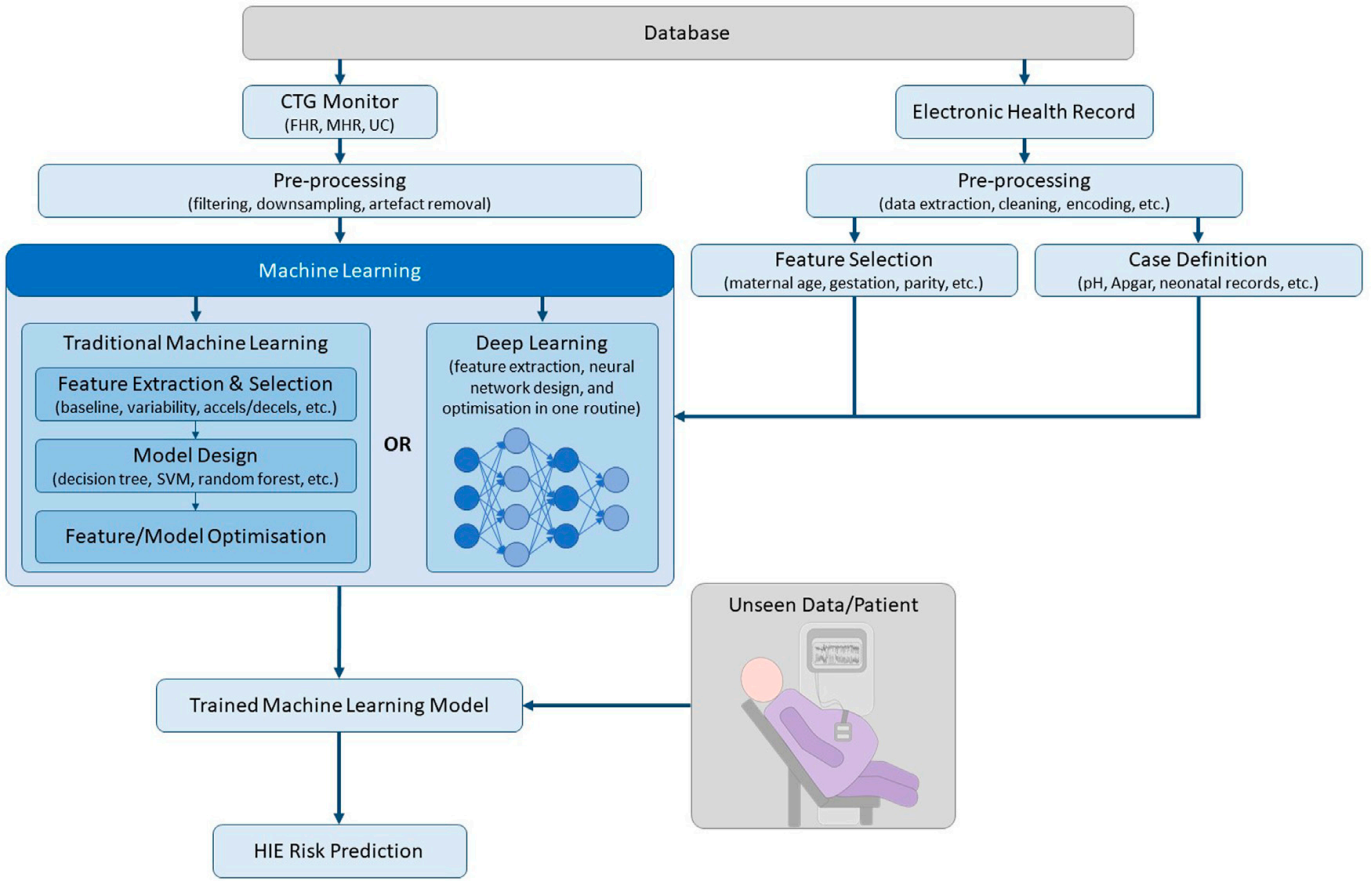
Pipeline of machine learning steps for CTG.

The aforementioned methods use traditional machine learning, which requires a feature extraction and selection stage before classification. Deep learning is a subset of machine learning, which uses a layered structure of calculations known as neural networks on unstructured data, whereby feature extraction and classification is performed in an optimised end-to-end routine, as depicted in Figure 1 (Garcia-Canadilla et al., 2020). While deep learning approaches require a relatively larger dataset, it offers the ability to learn complex features from the raw data, which may not be obvious to human experts. Deep Neural Networks (DNNs) were shown to outperform conventional machine learning algorithms, such as SVM and K-Means Clustering, for CTG classification on a database containing 162 normal cases and 162 abnormal cases (defined as pH < 7.20 and/or Apgar at 1 min <7) (Ogasawara et al., 2021). A multi-modal convolutional neural network (MCNN) architecture trained on over 35,000 patients was recently published (Petroziello et al., 2019). The MCNN takes input from the UC, FHR and signal quality measures. Its performance was assessed by measuring the percentage of interventions that were false positives and true positives. A retrospective analysis showed that current clinical practice resulted in a 15% false positive rate (FPR) and a 31% true positive rate (TPR), while the MCNN achieved a 14% FPR and a 50% TPR.

The RCT of the INFANT system concluded that including additional clinical information pertaining to labour could improve outcomes (Brocklehurst et al., 2017). Clinical information including maternal age, prior obstetric outcomes, thick meconium and uterine rupture were shown to be independent risk factors of severe neonatal acidosis (Maisonneuve et al., 2011). Similarly, results have shown that data-driven systems that use clinical risk factors result in improved classifier performance (Georgieva et al., 2017).

Table 1 summarises the above studies based on method, inputs, target, dataset size, and findings.

**TABLE 1:** Prior art comparison.

Author, year	ML methods	Input features	Target/labels	No. of patients	Type of study	Key finding
Brocklehurst et al. (2017)	Numerical algorithms and artificial neural network	Signal quality, baseline, variability, accels, decels and clinical data (dilation, analgesia, fetal blood sampling, growth restriction, placenta abruption and meconium)	Manually labelled CTG	47,000	RCT	Effective in identifying abnormal CTG, however clinical outcomes not improved
Nunes et al. (2017)		Contractions, accels, decels	Manually labelled CTG	7,320	RCT	Low rates of acidosis observed, however reduction in acidosis between the control arm and the interventional arm were not statistically significant
Ignatov and Lutomski (2016)		FHR, decels, FHR micro-fluctuations (extrema per minute, mean beat-to-beat variability, oscillation amplitudes)	Hypoxia (cord-artery blood ph < 7.20), acidemia (umbilical-artery blood pH < 7.05), intervention (caesarean or forceps)	720	RCT	Reduced risks observed for all targets in interventional arm
Warrick et al. (2009)	Support vector machine	Baseline, variability, impulse response function for decels and contractions	Base deficit (>12 mmol/L), death or HIE	213	Rtrspec. study	50% of pathological cases correctly detected with a false positive rate of 7.5%
Georgieva et al. (2014)		Decelerative capacity	Acidemia (pH < 7.05)	7,568	Rtrspec. study	AUC of 0.665 as a single feature in predicting acidemia
Doret et al. (2015)		Hurst parameter	Acidemia (pH < 7.05)	45	Case control study	AUC of 0.87 in predicting acidosis
Dash et al. (2014)	Generative models and Bayesian theory	FHR baseline, variability, accelerations, decelerations, FHR response to contractions in 4.5–30 mHz, variability in 30–1000 mHz band	Acidemia (ph < 7.15)	83	Rtrspec. study	0.817 TNR and 0.609 TPR
Ogasawara et al. (2021)	CNN	FHR	Acidemia (umbilical artery pH < 7.20) or Apgar at 1 min <7	324	Rtrspec. study	AUC of 0.73 with CNN, which was higher than traditional ML
Petroziello et al. (2019)	Multi-modal CNN	Signal quality, FHR, UC	Acidemia (pH < 7.05) and severe compromise (stillbirth, neonatal death, neonatal encephalopathy, NICU admission)	35,429	Rtrspec. study	Improved prediction of acidemia/compromise compared with clinical practice (14% FPR & 50% TPR versus 15% FPR & 31% TPR)
Georgieva et al. (2017)		Decelerative capacity and clinical data (presence of thick meconium or preeclampsia)	Acidemia (pH < 7.05) and severe compromise (stillbirth, neonatal death, neonatal encephalopathy, NICU admission)	22,790	Cohort study	Improved sensitivity and false-positive rate in detecting acidemia/compromise compared to clinical practice
Hoodbhoy et al. (2019)	XGBoost	21 features including basic quantitative values (max, min, median), STV, and number of fetal movements, decelerations and contractions	Manually labelled CTG	2,126	Rtrspec. study	Overall accuracy of 93%

## 3 Challenges in AI for CTG

### 3.1 Case Definition and Class Imbalance

CTG provides information on how the fetus is coping during labour, with the aim of allowing clinicians to detect non-reassuring fetal status so that adverse outcomes can be avoided through intervention. However, non-reassuring fetal status can result in a spectrum of outcomes, from a wholly unaffected fetus (due to a false positive CTG) to death (Gravett et al., 2016). Therefore, the question arises as to how a “control” patient versus a “pathological case” patient should be labelled in a machine learning architecture.

The incidence rate of HIE is 1-3 per 1,000 in high income countries (Kurinczuk et al., 2010). HIE is the primary condition that a CTG classifier should be trying to predict so that clinicians can intervene and prevent adverse outcome. However, this results in a significant class imbalance between normal and HIE classes, which leads to challenges from a machine learning perspective. At the higher range of 3 per 1,000, it would require over 30,000 deliveries to obtain a database with 100 HIE cases. Minority class oversampling techniques, such as Synthetic Minority Oversampling Technique (SMOTE), have been successfully used in CTG classification studies to introduce synthetic examples in the feature space (Spilka et al., 2013) (Hoodbhoy et al., 2019). However, a sufficient number of genuine cases are still required to use such techniques to synthesize examples. Similarly, weighted errors for misclassifying an example from the minority class has been used to rectify the class imbalance problem (Petroziello et al., 2019).

Due to the difficulty of acquiring a database with comprehensive NICU records and HIE diagnoses, proxy metrics are often used to label classes. There are many proxies for HIE, both objective (pH, base deficit, lactate, and transfer to NICU) and subjective (Apgar scores), with varying degrees of correlation to HIE. Metrics such as pH are generally used as indicators of poor outcome (Malin et al., 2010). However, there is literature that shows ambiguity in the correlation between pH and outcome (Yeh et al., 2012). Quite often, only the umbilical venous pH is measured or recorded, whereas the arterial pH can be significantly lower than the venous pH in babies exposed to a period of acute cord compression shortly before delivery (Westgate et al., 1994). As highlighted in Table 1, there is no consistency in the prior art as to what outcome, metric or combination of metrics are used to define a pathological case. A recent systematic review of intrapartum uterine activity and neonatal outcomes found that, of the 12 studies that met the inclusion criteria, 7 used pH as an individual outcome, Apgar scores and base excess were reported as individual outcomes in 4 studies and only 1 study reported neonatal encephalopathy as an outcome. No study examined long-term neurodevelopment as an outcome (Reynolds et al., 2020a). The Apgar score was not designed as a measure of birth asphyxia, and a recent cohort study including 85,076 infants concluded that although there is a close association between Apgar score and acidosis, Apgar score should not be used as a measure of birth asphyxia (Cnattingius et al., 2020).

### 3.2 Weak Labels Versus Expert Annotated Labels

As previously discussed, proxy metrics, such as pH, are often used as individual metrics to distinguish between normal and pathological outcome. CTU-UHB CTG database is a publicly available database hosted on Physionet, which is commonly used for research purposes (cited by over 150 papers) (Chudácek et al., 2014). The database includes 552 CTG recordings from 9,164 recordings acquired from one hospital over a three-year period. Of the 552 patients, 44 had a pH value less than 7.05, which is the threshold commonly used in literature to define pathological cases (Spilka et al., 2013). Annotation by three experts on the same database labelled 149 as normal CTG, 115 as pathological CTG and 275 as suspect CTG. This highlights the disparity between low pH and abnormal CTG (Spilka et al., 2013).

A major challenge with developing machine learning architectures based on proxies and neonatal outcomes is the fact that these labels are “weak.” The raw CTG in these cases are not labelled by event or by epoch. Instead, there is one overall label for the patient based on clinical metrics (i.e. pH < 7.05), regardless of the duration of the CTG abnormality, or the type of hypoxia. Different types of fetal hypoxia (acute, subacute, evolving, chronic) generally manifest in different forms in the CTG, and are associated with widely differing clinical events (Yatham et al., 2020). This introduces problems, as in an acute event (such as cord prolapse, uterine rupture, or acute cord compression) the CTG may only change during the event. Therefore, labelling an entire CTG record as fetal hypoxia may introduce noisy labels and misclassifications. This is particularly problematic if weak labels are being applied to short epochs (i.e. overlapping windows of 15–30 min segments), as there is a significant risk of introducing predominantly noisy labels, unless the fetal hypoxia is chronic and prevalent throughout the duration of the recording. Furthermore, studies have shown that not all infants diagnosed postnatally with HIE have evidence of intrapartum hypoxia in the CTG (using current human interpretation) (Yatham et al., 2020).

Machine learning architectures that use hand-labelled CTG at an event/epoch level by an expert annotator would result in stronger labels and, in theory, achieve improved performance. In light of this, an expert obstetrician has manually labelled the aforementioned CTB-UHB database, which has also been made publicly available to supplement the original database (Romagnoli et al., 2020). Several studies have obtained significantly high percentage agreements between algorithm and human labels (Reynolds et al., 2020b). However, introducing human labels may result in similar clinical outcomes to those observed in the prior RCTs, whereby high algorithm-human agreement is achieved but it is akin to adding a second evaluator with similar instructions. Similarly, multiple studies have shown inter-observer agreement for human CTG interpretation in the range of 30–50% (Yatham et al., 2020) (Hruban et al., 2015) (Rhöse et al., 2014). Therefore, there is a risk that human annotations may introduce human bias into the classification, given that expert use of CTG in general is still widely debated (Garcia-Canadilla et al., 2020).

Classification of CTG at an event level alone, without context of the labour progress and duration is not ideal, as features and patterns that may be considered non-reassuring in 1st stage of labour can be considered normal during the active 2nd stage of labour where contractions become more intense. As the end of the CTG often coincides with the time of birth, it is likely that relevant data pertaining to outcome would be most evident in the later stages of CTG. However, there is considerably more noise and motion artifacts in the later stage. Therefore, classifier performance can vary depending on the stage of labour. Studies have shown that the performance of features for classification of fetal compromise vary significantly as labour progresses (Spilka et al., 2014). As such, many studies in the literature omit 2^nd^ stage data, which may reduce the clinical usefulness of a decision support tool in practice (Spilka et al., 2016).

Having access to large databases, capable of training a deep learning model may help resolve this issue, as the feature extraction and classification process could be completed in an optimized routine. The variation in model performance based on the stage of labour was demonstrated in (Petroziello et al., 2019) using a MCNN trained on 35,000 CTGs. The performance of the MCNN trained on the last 60 min of 1st stage was 0.65 AUC, while the same MCNN model trained on the last 30 min of 2nd stage was 0.71 AUC. The best performance of 0.77 AUC was achieved by training on the last 60 min of CTG, regardless of stage (Petroziello et al., 2019).

## 4 Discussion

Previous feature-based approaches to automated CTG interpretation that closely follow established CTG clinical guidelines achieve high inter-observer agreement with human interpretation. However, they do not result in improvements in clinical outcomes. The findings of these studies suggest that developing systems to mimic existing guidelines and human interpretation will not improve outcomes. More recent methods, facilitated by more computing power, comprehensive electronic health records, and access to larger datasets have resulted in promising developments. However, these approaches are yet to be validated in a RCT.

The major challenges identified in developing robust AI for CTG interpretation are centred around case definition, labelling and class imbalance, which are inherently linked. The table demonstrates the variability in case definition across the prior art, with many using proxy metrics, such as pH, to label cases as healthy versus HIE. At an incidence rate of 1-3 per 1,000 births, class imbalance is a major concern, and perhaps an anomaly detection approach may be best suited.

While accurately detecting non-reassuring CTG patterns is important, it is not the primary challenge. The primary challenge is determining whether non-reassuring CTG patterns require intervention or not based on the progression of labour and on the risk profile of the mother. Our previous work has demonstrated that improvements in classification performance are achievable by adding both clinical variables (such as gestation, parity and hypertension), as well as duration of labour stages (O'Sullivan et al., 2021). The importance of accurate medical records is critical to the clinical decision-making process. Pre-existing maternal medical conditions such as chronic hypertension, and underlying conditions such as intrauterine growth restriction, can render the utero-placental system more vulnerable to hypoxia during labour (Scheidegger et al., 2019). The clinical team need to consider the risk profile of a pregnancy to aid their assessment of a fetus’ tolerance to labour and need to be vigilant for any non-reassuring patterns in high-risk pregnancies. Providing a decision support tool that is developed without consideration of these personalised risk factors and their relationship to neonatal outcomes may result in an increase in unnecessary C-sections and operative delivery rates.

To conclude, there is significant scope and promise for decision support tools in the area of CTG, as demonstrated by prior art. We believe that accurate case definition and segmentation of the data, combined with the inclusion of pre-existing clinical variables and labour progression data will facilitate the development of an explainable artificial intelligence decision support tool.
